# Popular Science Department

**Published:** 1882-09

**Authors:** 


### POPULAR SCIENCE DEPARTMENT.

### PROPERTIES OF NITRO-GLYCERINE.

It has a sweet, aromatic, pungent taste, and possesses the very peculiar property of causing an extremely violent headache when placed in a small quantity upon the tongue, or any other portion of the skin, particularly the wrist. It has long been employed by homœpathic practitioners as a remedy in certain kinds of headaches. In those who work much with it, the tendency to headache is generally overcome, though not always. It freezes at about 40° Fahr., becoming a white half-crystallized mass, which must be melted by the application of water at a temperature of 100° Fahr. If perfectly pure—that is, if the washing has been so complete as to remove all traces of the acid—it can be kept for an indefinite period of time; and, while many cases of spontaneous combustion have occurred in impure specimens, there has never been known such an instance, where the proper care has been given to all the details of the manufacture.

When pure, nitro-glycerine is not very sensitive to friction, or even to moderate percussion; if a small quantity be placed on an anvil and struck with a hammer, that portion which is touched explodes sharply, but so quickly as to drive away the other particles; if, however, it were even slightly confined, so that none could escape, it would all explode or detonate. It must be fired by a fuse containing fulminate of mercury (the compound used in percussion caps), not being either readily or certainly fired by gunpowder, the shock of the latter not being sufficiently quick or sharp to detonate the nitro-glycerine. It is highly probable that in this case, as in that of other high explosives, the vibrations set up by the fulminate (which is not stronger than gunpowder) are of just such a character to find an answering chord, so to speak, in the explosive, so that the desired effect is produced. This would seem to be a correct theory, for it is not always the most powerful explosive which most readily causes the explosion of another body. For instance, although nitro-glycerine is much more powerful than fulminate of mercury, yet seventy grains of it will not explode gun-cotton, while fifteen grains of the weaker fulminate will readily do so. The fuse generally used, then, for firing nitro-glycerine, is composed of from fifteen to twenty-five grains of fulminate, and this quantity is sufficient to detonate a large mass as well as a small one.

If flame be applied to nitro-glycerine it will not explode, but burn with comparative sluggishness. When frozen it is very difficult and uncertain of firing. If the material be perfectly pure it forms, upon detonation, a volume of gases nearly thirteen hundred times as great as that of the original liquid ; these gases are also further expanded, by the heat developed, to a theoretical (though not practical) volume ten thousand times as great as that of the charge. Practically speaking, the forces exerted by gunpowder and nitro-glycerine are in the proportion of one to eight.—*From "Explosions and Explosives," by* ALLAN D. BROWN, *in Popular Science Monthly for October.*

### THE CHIN AS AN INDEX OF CHARACTER.

If the experience of mankind is competent to interpret facial indications, some of the "propensities" and "perceptives" which Spurzheim lodges in the back rooms of his pan-sensorium must have a *penchant* for changing their quarters. "Firmness," for instance, which he locates in the posterior part of the upper head, undoubtedly manifests itself in the prominence of the *chin.* "Draw a face in profile," says Winckelman, "and observe how timidity or its opposite can be expressed by the shape of the lower jaw. Let the chin be receding, and your profile can be made to express pusillanimity and feebleness of character, even to the degree of imbecility. Then, without changing any upper line of the profile, combine it with a prominent chin, and it will exhibit firmness. Exaggerate the prominence, and you can intensify that expression to one of obstinacy and ferocity. That such contrasts are less striking in living faces is owing to the circumstance that we take in the expression of all features at a single glance, without analyzing the complex effect."

Have we not here a positive criterion, a rule without an exception? Does it not occur to us, on afterthought, that *all* warlike, aggressive nations have such projecting chins, while the weak or degenerate ones are more or less chinless? In their classification of the North American aborigines the Spaniards distinguish between *Indios mansos* and *Indios bravos* (tame and savage Indians). The former comprise the different agricultural tribes of Central and South America, ignorant but harmless creatures, who subsist on a vegetable diet; the latter, the carnivorous savages of the North, who divided their time between hunting and warfare. In their physical characteristics these various tribes of the American autochthones could hardly be distinguished, if it were not for a slight variations in the color of their skins and a very marked difference in the shape of their *chins*. Our redskins *have* chins, though they cannot emulate those of the Indio-Germanic race; the Indians of Mexico and South America have none. In the profile of a vegetarian Indio from the neighborhood of Vera Cruz, the lower jaw recedes in a sharp line from the mouth to the throat, so that his nose, though not excessive in size, becomes ridiculously prominent. Obstinacy with a projecting chin and shrinking timidity with a receding one are here strongly contrasted, and the study of individual faces proves Winkelmann's rule to be almost, if not altogether, infallible. Can Professor Fowler point out a corresponding difference in the shape of the posterior skull? "Amativeness," too, may or may not affect the bones above the nape, but Theoprastus, Galen, Della Porta, Lavater, Dr. Redfield and all portrait painters, agree that it *is* disclosed by the *eyelids*, especially the lower ones.

Lavater and other critical students of the human face have shown the fallacy of some popular notions; for instance, the connection of a high forehead with superior intelligence—but also that generally received opinions differ less in different nations and ages than should be supposed; and it is surprising of what minute symptoms even the ancient nations have taken cognizance.—DR. FELIX L. OSWALD *in Popular Science Monthly for October*.

### AMERICAN INVENTION IN THE EGYPTIAN WAR.

Among the supplies for the British army in Egypt mention is made of driving apparatus, tubing, and pumps for two hundred "Abyssinian wells," by which name American drive wells are known in England, from the circumstance that they were first used by the British army in the Abyssinian war. It is estimated that two hundred wells of the capacity ordered will furnish from two to three million gallons of water a day, and make the army independent of the surface water sources of the country. Seeing that the fresh water canals are largely in the control of Arabi, the success of the invasion may be largely contingent upon the ability which drive wells give of obtaining water anywhere in the desert.

This, however, does not exhaust the indebtedness of the British forces to American inventors. The great war ships of England are supplied with the Brush electric lamps invented at Cleveland ; and, as every reader will recall, it was by means of the powerful lights of the fleet that Arabi's attempts to strengthen the forts about Alexandria, under cover of night and contrary to agreement, were detected and frustrated. After the bombardment began the electric lights played a not less important part in directing the movements of the ships at night, in guarding against surprises, and in watching the movements of the enemy on shore.

During the bombardment the most effective service was done by turreted vessels ; and the revolving turret is an American invention.

The machine gun, another American invention, has proved an extremely efficient arm for the invading forces. One vessel fired 6,000 pounds of shot from Gatling guns the first day of the bombardment. A handful of marines, with guns of this type, were able to disperse the Alexandrian "looters" and restore order in the afflicted city, where many times their number would have failed without such aid.

In the subsequent skirmishing with Arabi's troops about Alexandria, later in the capture of Shaluf and other fortified places along the Suez Canal, the same guns on the gunboats and on shore have been in constant use.

It is not so well known that the small arms of the British soldiers are but slightly modified American guns, made with machinery patterned after that developed in the shops of Springfield, Mass. The system of fixed ammunition for small arms also, and the machines by which such cartridges are made, are all of American origin.—*Scientific American.*

### ACCIDENT WITH ELECTRIC LIGHT WIRES.

The first fatal accident with electric light wires in this city occurred October 4, the victim being an experienced line man in the employ of the Brush Electric Light Company. He was engaged in splicing a "live" wire to increase its length so that it could be transferred to another and higher pole. To do this without interrupting the current the splice had to be inserted as a loop around the point to be cut; and in making the loop connections the insulating material of the wire had to be scraped away to secure contact of the naked wires. The rule of such work is to complete one connection before beginning the other, and to complete both connections before cutting the wire, exercising meantime the utmost care to avoid touching the wires so as to allow any portion of the body to be brought into the circuit of the electric current or any part of it. By some slip or other unexplained mishap, the line man failed to properly observe these precautions, and the failure cost him his life. He was caught by the wires in such a way that he did not fall to the ground, though he was unconscious from the moment he received the shock. The fact that he did not die instantly is thought to prove only part of the current passed through his body. The palms of both hands were burned. The wire from which the fatal shock was received was carrying electricity for forty lights of 2,000 candle power each.—*Scientific American.*

### INDUSTRIAL EDUCATION IN THE PUPLIC SCHOOLS.

There is a growing feeling among the students of industrial problems that our whole conception of education in general, and of industrial training in particular, needs revision and enlargement. This feeling is based upon such easily observed facts as the following:

1. Paupers are on the increase.

2. Our schools too often educate their pupils out of harmony with their environment, thus justifying the charge that education (falsely so called) unfits its possessors for useful industry.

3. The simpler and less important positions in the world's workshop are as a rule greatly overcrowded, while in the upper stories there is a vast amount of unoccupied space.

4. The work done in the lower stories is often exceedingly shabby.

5. Many who aspire to the upper stories fail to enter—or, if they apparently enter, soon end in failure.

6. The chosen few who truly enter, and build up magnificent industrial fabrics, with the splendid fortunes which such fabrics imply, fail to educate their children to carry on their good work, or to do work of similar value in some other department of useful industry.

7. A whole community of prosperous workmen may be well-nigh reduced to beggary by the incoming of some new invention, or by change in the fortunes or tastes of consumers.

8. When old industries are swept away, and new ones established on the wrecks, there is usually little power on the part of workmen to adapt themselves to the new conditions.

9. The relentless law of the survival of the shrewdest and most unscrupulous, instead of the Christian law of mutual consideration and co-operation, too generally prevails among individuals and all kinds of human organizations.

That all education should be industrial, and that everybody should be industrially educated, we believe to be a perfectly tenable proposition.—PROFESSOR H. H. STRAIGHT, *in Popular Science Monthly for October.*

### WHAT ARE CLOUDS?

Though the clouds are such familiar objects, very little is known about them, and the processes by which they are formed and give back their moisture to the earth are unsolved mysteries.

They cannot be classified as belonging to the solid, fluid, or gaseous form of matter. Yet they are defined as being "a collection of watery particles in the state of *vapor*, suspended in the air." If they are ordinary vapor, they must be governed by the laws which affect vapors. Brande defines *vapor* thus: "When liquids and certain solids are heated, they become converted into elastic fluids or vapors, which differ from gases in this respect, that they are not under common circumstances permanently elastic, but resume the liquid or solid form when cooled down to ordinary temperature." According to this definition, clouds cannot be composed of *ordinary* vapor, for under all conditions their temperature must be below the condensing point of water-vapor.

At the elevations at which clouds are often seen, they are in the regions of perpetual congelation; and as they float above the highest mountains they must be exposed, even in the sunshine, and certainly in the night, when the solar heat is not poured upon them, to temperatures colder than those of the frigid zones.—C. Morfit, *in Popular Science Monthly for October.*

### A DISCOVERY OF A GRAND HALL NEAR THE PANTHEON, AT ROME.

A grand hall, exceeding in length the full interior of the Pantheon, and supposed to be the vestibule of the Pantheon itself, or rather, a connecting hall between the Pantheon and the Baths of Agrippa, has been recently explored. This hall measures 140 feet in length, 50 feet in width, adorned with eight splendid fluted columns of Phrygian and Numidian marble. Within this hall is a niche, where is a pedestal 12 feet wide by 11 feet high, large enough for a colossal group of sculpture. It is supposed that within this hall stood the celebrated bronze "Athlete" statute, which Agrippa brought from Greece, and placed in the portico of his warm baths.

